# Neutrophil-to-lymphocyte ratio is associated with increased cerebral blood flow velocity in acute bacterial meningitis

**DOI:** 10.1038/s41598-021-90816-0

**Published:** 2021-05-31

**Authors:** Antje Giede-Jeppe, Selim Atay, Julia Koehn, Anne Mrochen, Hannes Luecking, Philip Hoelter, Bastian Volbers, Hagen B. Huttner, Lena Hueske, Tobias Bobinger

**Affiliations:** 1 Department of Neurology, University Hospital Erlangen, University of Erlangen-Nuremberg, Schwabachanlage 6, 91054 Erlangen, Germany; 2 Department of Neuroradiology, University of Erlangen-Nuremberg, Erlangen, Germany; 3 Department of Neurology, University Hospital Gießen, Gießen, Germany; 4 Neurological Hospital for Parkinson’s Disease, Beelitz‑Heilstaetten, Germany

**Keywords:** Medical research, Outcomes research, Meningitis

## Abstract

In community-acquired bacterial meningitis (CABM) intracranial vascular alterations are devastating complications which are triggered by neuroinflammation and result in worse clinical outcome. The Neutrophil-to-Lymphocyte ratio (NLR) represents a reliable parameter of the inflammatory response. In this study we analyzed the association between NLR and elevated cerebral blood flow velocity (CBFv) in CABM-patients. This study included all (CABM)-patients admitted to a German tertiary center between 2006 and 2016. Patients’ demographics, in-hospital measures, neuroradiological data and clinical outcome were retrieved from institutional databases. CBFv was assessed by transcranial doppler (TCD). Patients’, radiological and laboratory characteristics were compared between patients with/without elevated CBFv. Multivariate-analysis investigated parameters independently associated with elevated CBFv. Receiver operating characteristic(ROC-)curve analysis was undertaken to identify the best cut-off for NLR to discriminate between increased CBFv. 108 patients with CABM were identified. 27.8% (30/108) showed elevated CBFv. Patients with elevated CBFv and normal CBFv, respectively had a worse clinical status on admission (Glasgow Coma Scale: 12 [9–14] vs. 14 [11–15]; *p* = 0.005) and required more often intensive care (30/30 [100.0%] vs. 63/78 [80.8%]; *p* = 0.01).The causative pathogen was *S. pneumoniae* in 70%. Patients with elevated CBFv developed more often cerebrovascular complications with delayed cerebral ischemia (DCI) within hospital stay (*p* = 0.031). A significantly higher admission-NLR was observed in patients with elevated CBFv (median [IQR]: elevated CBFv:24.0 [20.4–30.2] vs. normal CBFv:13.5 [8.4–19.5]; *p* < 0.001). Multivariate analysis, revealed NLR to be significantly associated with increased CBFv (Odds ratio [95%CI] 1.042 [1.003–1.084]; *p* = 0.036). ROC-analysis identified a NLR of 20.9 as best cut-off value to discriminate between elevated CBFv (AUC = 0.713, *p* < 0.0001, Youden's Index = 0.441;elevated CBFv: NLR ≥ 20.9 19/30[63.5%] vs. normal CBFv: NLR > 20.9 15/78[19.2%]; *p* < 0.001). Intracranial vascular complications are common among CABM-patients and are a risk factor for unfavorable outcome at discharge. Elevated NLR is independently associated with high CBFv and may be useful in predicting patients’ prognosis.

## Introduction

Bacterial meningitis is a severe infection of the central nervous system^1,2^. In the development of bacterial meningitis the host inflammatory response plays an important role resulting in activation of both non cellular and cellular components of the immune system^3,4^. In the circulation neutrophils constitute the dominant cell type mediating the earliest innate immune responses getting to the site of infection^5^. Massive neutrophil recruitment is required across the blood–brain-barrier (BBB) to evoke a strong inflammatory response to react to the microorganisms at the bacterial infection site^4^. Activated immune cells within the brain, such as microglia, astrocytes and infiltrating leukocytes amplify the cascade of pro-inflammatory cytokines and cytotoxic agents. This activation of the immune response with subsequent rapid influx of leukocytes into the brain also causes adverse effects for the host as e.g. intracranial vascular alterations^6^. Intracranial vascular alterations may result in damage to cortical and subcortical structures^7,8^ which further result in edema, hydrocephalus and increased intracranial pressure^9^. Therefore they represent a devastating complication leading to unfavorable outcome and increased morbidity^10^. The inflammatory process in bacterial meningitis can be monitored by numerous biochemical markers^11,12^. The Neutrophil-to-Lymphocyte ratio (NLR) represents information on both the innate and adaptive immune system and is a reliable parameter for the general immune response to various stimuli. Neutrophil-to-Lymphocyte ratio (NLR), calculated by absolute neutrophil count divided by absolute lymphocyte count, is easy to perform in routine practice and cost-effective. Neutrophil-to-Lymphocyte ratio (NLR) has proven its prognostic value in cerebrovascular^13,14^ and inflammatory diseases^15^, several types of tumors^16–18^, infections^19,20^ and also in the differential diagnosis between viral and bacterial meningitis^21,22^. This study aimed to analyze the association between NLR and elevated cerebral blood flow velocity (CBFv) which represents a devastating complication in bacterial meningitis.

## Methods

### Patients und Inclusion criteria

**Figure 1:**
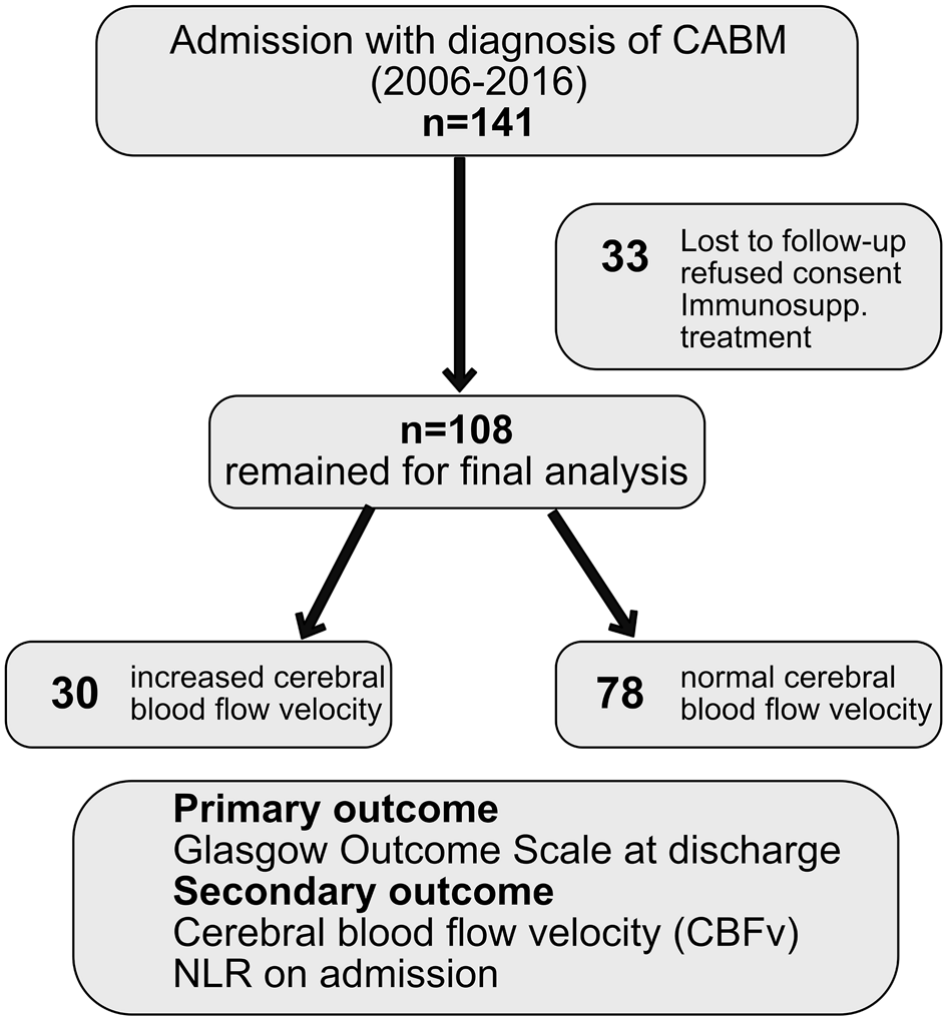
Flowchart of patients. 141 patients with community-acquired bacterial meningitis were identified during the study period. After exclusion of 33 patients 108 patients remained for further analysis. Patients were dichotomized according to increased cerebral blood flow velocity (N = 30) and normal cerebral blood flow velocity (N = 78). Abbreviations: *CABM* community acquired bacterial meningitis, *CBFv* cerebral blood flow velocity, *GOS* Glasgow outcome scale (range, 5 no or mild deficit, to 1, death), *NLR* Neutrophil-to-Lymphocyte ratio.

All consecutive patients with diagnosis of CABM admitted to the Department of Neurology, University Hospital Erlangen, Germany were included in a prospective longitudinal institutional database, which was approved by the institutional ethics committee. Out of this database, all patients (N = 141) admitted between 2006 and 2016 for treatment of CABM have been recruited to this study. We excluded patients receiving permanent immunomodulatory treatment (e.g. corticosteroids, methotrexate, cytostatic drugs and biologicals) on admission (n = 14). Patients without follow-up data or refused consent were also excluded (n = 19). 108 patients remained for final analysis (Fig. 1).

### Data collection

Data of all patients were retrieved from our institutional prospective database: patients' history (hypertension, diabetes mellitus, alcohol abuse or other comorbidities), GCS (Glasgow Coma Scale) on admission, clinical symptoms on admission, laboratory findings on admission (cerebrospinal fluid and blood work, causative pathogen), clinical course, outcome and neurologic findings at discharge, and treatment.

Arterial vascular alterations and incidence of cerebral ischemia were assessed using computed tomography imaging (CT-scan, (SIEMENS Somatom Volume Zoom, Somatom Sensation 64, Somatom Definition AS+; Siemens Healthcare, Forchheim, Germany) or magnetic resonance imaging (MRI) (SIEMENS Magnetom Sonata 1.5T, Magnetom Aera 1.5T, Siemens Healthcare, Erlangen, Germany).

NLR was calculated by dividing the absolute neutrophil count by the absolute lymphocyte count (NLR days 1, 2, 3, 4, 5, 6, 7, 8).

### Diagnosis of acute bacterial meningitis (ABM)

The diagnosis of acute bacterial meningitis was confirmed by identification of the causative pathogen in cerebrospinal fluid and/or blood via microscopic Gram stain, CSF/blood cultured isolates and PCR on CSF^23^. In case the causative pathogen could not be identified bacterial meningitis was diagnosed by typical CSF findings as mainly granulocytic CSF white blood cell count greater than 1000 cells/μl, an increase of CSF protein of more than 100 mg/dl, and a CSF/serum glucose ratio less than 0.3 and/or presence of clinical symptoms as fever, neck stiffness, headache, impaired consciousness^24^. CSF lactate level was measured using standard enzymatic test.

### Detection of increased cerebral blood flow velocity

Presence of vascular alterations was assessed by TCD as a routine clinical practice termed as cerebral blood flow velocity (CBFv) of the anterior cerebral artery (ACA), middle cerebral artery (MCA), internal cerebral artery (ICA), posterior cerebral artery (PCA), and the basilar artery (BA). Systolic CBFv greater than 150 cm/s were considered increased^25,26^.

### Outcome

Outcome at discharge was evaluated according to the Glasgow Outcome Scale (1–5) by two physicians, trained and certified for data collection: A score of 1 on this scale indicates death; a score of 2, a vegetative state; a score of 3, severe disability (the patient is not able to live independently but can follow commands); a score of 4, moderate disability (the patient is capable of living independently but unable to return to work); and a score of 5, mild or no disability (the patient is able to return to work). A favorable outcome was defined as a score of 5, and an unfavorable outcome as a score of 1–4. The Glasgow Outcome Scale is a well-validated instrument with high interobserver agreement^27,28^. Long-term outcome was evaluated using the modified Rankin Scale (mRS) 3 and 12 months after onset. Favorable outcome representing an independent clinical status was defined as mRS score of 0–2, unfavorable outcome as mRS score of 3–6^29^.

### Statistical analysis

Statistical analysis was performed using SPSS 22.0 (IBM Analytics, Armonk, New York, United States) and GraphPad Prism 8 (GraphPad Software, San Diego, California, United States). Categorial variables were presented as frequency and percentage, Pearson chi square and Fisher’s exact test were used to compare between these groups. For continuous variables, the Kolmogorov–Smirnov test was used to test the distribution of data. If data showed normal distribution, data was presented with mean ± SD and the Student t test was used for analysis. Data lacking normal distribution, median and interquartile range were shown and the Mann–Whitney U test was used for comparison. Significance level was set at α = 0.05. All parameters showing a statistical trend (*p* < 0.1) were included in a multivariate model to identify parameters independently associated with elevated cerebral blood flow. A Receiver Operating Characteristic (ROC) curve and Youden’s J statistic was used to determine the cut-off value for NLR^30^. Then, patients were dichotomized according to the identified cut-off value.

### Ethics approval and consent to participate

All procedures involving human participants were in accordance with the ethical standards of the institutional research committee of the University of Erlangen-Nuremberg and with the 1964 Helsinki declaration and its later amendments. The Institutional Ethics Committee of the University of Erlangen-Nuremberg had approved the study protocol (304_16 B).

### Informed consent

Informed consent was obtained from all subjects.

## Results

### Patient characteristics

Overall 108 patients with CABM remained for final analysis (Fig. 1). The overall cohort of patients was 59 ± 16 years old. 51.9% (56/108) were male. More than half of the patients presented with clinical signs suggestive of bacterial meningitis as fever 57.4% (62/108), headache 51.9% (56/108) and meningism 63.9% (69/108). Only 27.8% (30/108) of these patients showed further neurological deficits. 74.1% (80/108) received dexamethasone on admission*.* In 18/108 patients (16.7%) no pathogen was detected (Supplementary Table 1).

### Vascular complications

30/108 (27.8%) patients developed elevated cerebral blood flow velocity within 4 (3–5) days after admission. Patients with elevated CBFv versus normal CBFv: had a worse clinical status on admission (median [IQR]: Glasgow Coma Scale 12 [9–14] vs. 14 [11–15]; *p* = 0.005, neurologic deficit 13/30 [43.3%] vs. 17/78 [21.8%]; *p* = 0.025), required more often intensive care (30/30 [100%] vs. 63/78 [80.8%]; *p* = 0.010) with need of osmotherapy (13/30 [43.3%] vs. 6/78 [7.7%]; *p* < 0.001), therapy with nimodipine (23/30 [76.7%] vs. 3/78 [3.9%]; *p* < 0.001) and/or catecholamines (22/30 [73.3%] vs. 31/78 [39.7%]; *p* = 0.002) at a dedicated intensive care unit. They needed longer ventilation (167 h [45–510] vs. 0 h [0–206]; *p* = 0.001) because of reduced consciousness. In patients with increased CBFv CSF analysis confirmed typical abnormalities as polymorphonuclear leukocytosis, decreased glucose concentration, and increased protein concentration. Whereas only protein concentrations were significantly increased (2.910 g/L [1.491–4.287] vs. 1.501 g/L [0.629–3.038]; *p* = 0.009).The most common causative microorganism was *Streptococcus pneumoniae* (70% [21/30] vs. 26.9% [21/78]; *p* < 0.001) identified by CSF (28/30 [93.3%] vs. 51/78 [65.4%]; *p* = 0.003). In 3.3% (1/30 (3.3%) vs. 17/78 (21.8%), *p* = 0.021) no causative pathogen was identified. These patients showed a higher rate of infectious complications (sepsis: 17/30 [56.7%] vs. 30/78 [38.5%]; *p* = 0.087). Cerebral infarctions during hospital stay occurred more frequently (9/27 [33.3%] vs. 3/30 [10.0%]; *p* = 0.031) translating into an unfavorable outcome at discharge (Glasgow Outcome Scale 3 [3, 4] vs. 4 [3–5]; *p* = 0.028, Table 1). Infarcts morphologically due to cerebral vasospasm were predominantly located in the vascular territory of the anterior and/or middle cerebral artery (Supplementary Table 2).

**Table 1:** Baseline characteristics, laboratory data, in-hospital measures and outcome parameters for all patients with community-acquired bacterial meningitis developing increased cerebral blood flow velocity.

Meningitis		Increased cerebral blood flow velocity (N = 30)	Normal cerebral blood flow velocity (N = 78)	*p* value
Gender (♀)^b^		23 (76.7%)	33 (42.3%)	**0.001**
**Prior medical history—admission status—in hospital measures**				
Premorbid mRS^a^		0 (0–1)	0 (0–1)	0.278
Alcohol abuse^b^		9 (30.0%)	6 (7.7%)	**0.003**
Neurologic deficit^b^		13 (43.3%)	17 (21.8%)	**0.025**
Glasgow Coma Scale^a^		12 (9–14)	14 (11–15)	**0.005**
Length of ventilation (LOV) (h)^a^		167 (45–510)	0 (0–206)	**0.001**
Dexamethasone on admission^b^		26 (86.7%)	54 (69.2%)	*0.064*
Stay on Neurointensive Care Unit^b^		30 (100.0%)	63 (80.8%)	**0.010**
Osmotherapy^b^		13 (43.3%)	6 (7.7%)	** < 0.001**
Nimodipine therapy^b^		23 (76.7%)	3 (3.9%)	** < 0.001**
Need of Catecholamines^b^		22 (73.3%)	31 (39.7%)	**0.002**
Temperature on admission (°C)		38.3 (37.5–39.2)	38.8 (38.1–39.4)	*0.064*
Sepsis^b^		17 (56.7%)	30 (38.5%)	*0.087*
**Laboratory values on admission**				
*First spinal tap*				
Leucocytes (× 10^6^/L)^a^		2328 (187–5788)	1020 (216–5104)	0.661
Erythrocytes (× 10^6^/L)^a^		234 (5–555)	27 (1–160)	**0.032**
Proteine (g/L) ^a^		2.910 (1.491–4.287)	1.501 (0.629–3.038)	**0.009**
Glucose (mmol/L)^a^		1.28 (0–3.61)	2.28 (0.22–3.44)	0.676
Lactate (mmol/L)^a^		9.8 (5.2–16.9)	7.1 (3.5–13.8)	0.178
Causative pathogen identified by blood^b^		17 (56.7%)	31 (39.7%)	0.113
Causative pathogen identified by CSF^b^		28 (93.3%)	51 (65.4%)	**0.003**
Causative pathogen identified by CSF via PCR^b^		14 (46.7%)	28 (35.9%)	0.304
No causative pathogen identified^b^		1 (3.3%)	17 (21.8%)	**0.021**
**Most frequent causative pathogen**				
*S. pneumoniae*^b^		21 (70.0%)	21 (26.9%)	** < 0.001**
**Serum**				
Neutrophil–Lymphocyte-Ratio^a^		24.0 (20.4–30.2)	13.5 (8.4–19.5)	** < 0.001**
Thrombocytes (10^9^/L)^c^		172.7 ± 62	208.0 ± 98	*0.078*
Granulocytes (10^9^/L)^a^		15.6 (13.7–20.8)	12.1 (8.1–15.4)	**0.017**
Lymphocytes (10^9^/L)^a^		0.7 (0.6–1.0)	0.9 (0.8–1.3)	**0.024**
**Radiological data**				
*First CT on admission*				
Abscess^b^		1 (3.3%)	2 (2.6%)	0.627
Ischemia^b^		2 (6.7%)	2 (2.6%)	0.308
Obstructive hydrocephalus^b^		4 (13.3%)	1 (1.3%)	**0.020**
**CT within hospital stay- follow up (57/108 patients received a second cerebral imaging)**				
Abscess^b^		3/27 (11.1%)	3/30 (10.0%)	0.613
Ischemia^b^		9/27 (33.3%)	3/30 (10.0%)	**0.031**
Obstructive hydrocephalus^b^		3/27 (11.1%)	1/30 (3.3%)	0.266
**Discharge status**				
Symptomatic epilepsy^b^		6 (20.0%)	3 (3.8%)	**0.013**
Glasgow Outcome Scale (GOS)		3 (3–4)	4 (3–5)	**0.028**
LOV^b^		167 (45–510)	0 (0–206)	**0.001**
Length of stay (d)^a^		22 (18–26)	15 (9–20)	** < 0.001**
mRS at discharge ^a^		4 (2–5)	1 (1–4)	**0.020**
mRS at 3 months ^a^		2 (2–4)	2 (1–3)	0.166
mRS at 12 months ^a^		2 (1–4)	1 (0–3)	0.238

^a^Median (IQR), ^b^No. (%), ^c^Mean ± standard deviation.

Abbreviations: *CBFv* cerebral blood flow velocity, *GOS* Glasgow outcome scale (range, 5 no or mild deficit, to 1, death), *mRS* modified Rankin Scale (range 0, no deficit, to 6, death), *NIHSS Scale* National Institutes of Health Stroke Scale, *NLR* Neutrophil-to-Lymphocyte ratio, *IQR* interquartile range, *CSF* Cerebrospinal fluid.

*p*-value: Boldface type indicates statictical significance, findings showing a statistical trend are expressed in italics.

Further, a significantly higher NLR on admission was observed in patients with elevated blood flow velocity (median [IQR]: elevated CBFv: 24.0 [20.4–30.2] vs. normal CBFv: 13.5 [8.4–19.5]; *p* < 0.001). Parameters with tendency for significance (*p* < 0.1) in univariate testing were included into a multivariable model. In this model NLR levels on admission were associated with increased cerebral blood flow velocity (1.042 (1.003–1.084); *p* = 0.036; Table 2) as well as need of osmotherapy (*p* = 0.026). Need of catecholamine therapy (*p* = 0.561), clinical status on admission (*p* = 0.116) and need of ventilation (*p* = 0.178) were not significantly associated with increased CBFv.

**Table 2:** Multivariate analysis of parameters associated with increased cerebral blood flow velocity.

Parameters	95% CI	*p* value
NLR D1	1.042 (1.003–1.084)	**0.036**
Catecholamine therapy	0.634 (0.137–2.944)	0.561
Osmotherapy	0.181 (0.040–0.819)	**0.026**
Neurologic deficit on admission	2.693 (0.784–9.254)	0.116
Ventilation	3.001 (0.606–14.857)	0.178

Multivariable regression analysis was calculated for the association with increased cerebral blood flow velocity. Only parameters showing a statistical trend (*p* < 0.1) in prior univariate testing were included in the multivariable model. For each parameter risk ratio and 95% confidence interval are provided. Significant findings are expressed in bold.

*NLR* Neutrophil-to-Lymphocyte ratio, *95% CI* Confidence Interval.

### Association of Neutrophil-to-Lymphocyte ratio with increased cerebral blood flow velocity

**Figure 2:**
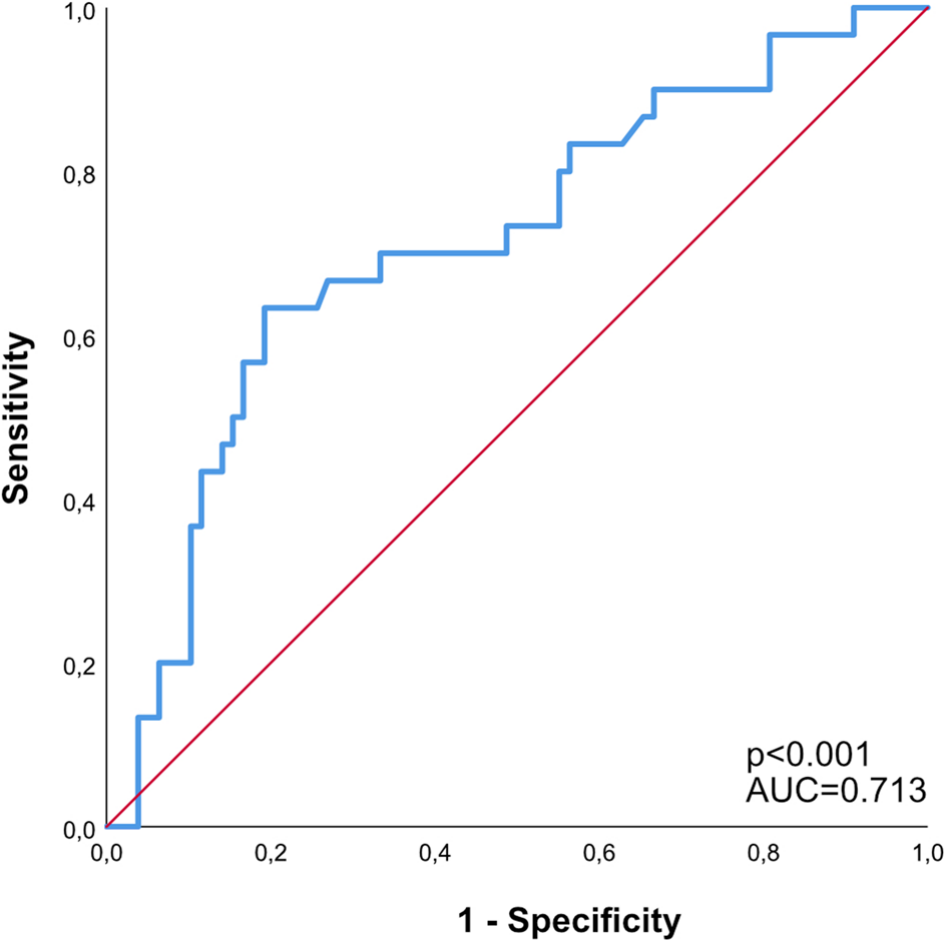
Association of Neutrophil-to-Lymphocyte ratio (NLR) with increased cerebral blood flow velocity. Receiver operating characteristic (ROC)—curve for prediction of increased cerebral blood flow velocity. ROC plot demonstrated the AUC for increased cerebral blood flow velocity (AUC = 0.713; *p* < 0.001, Youden’s index = 0.441; sensitivity, 63.3%; specificity, 80.8%). The cut-off value was detected at 20.9.

**Figure 3:**
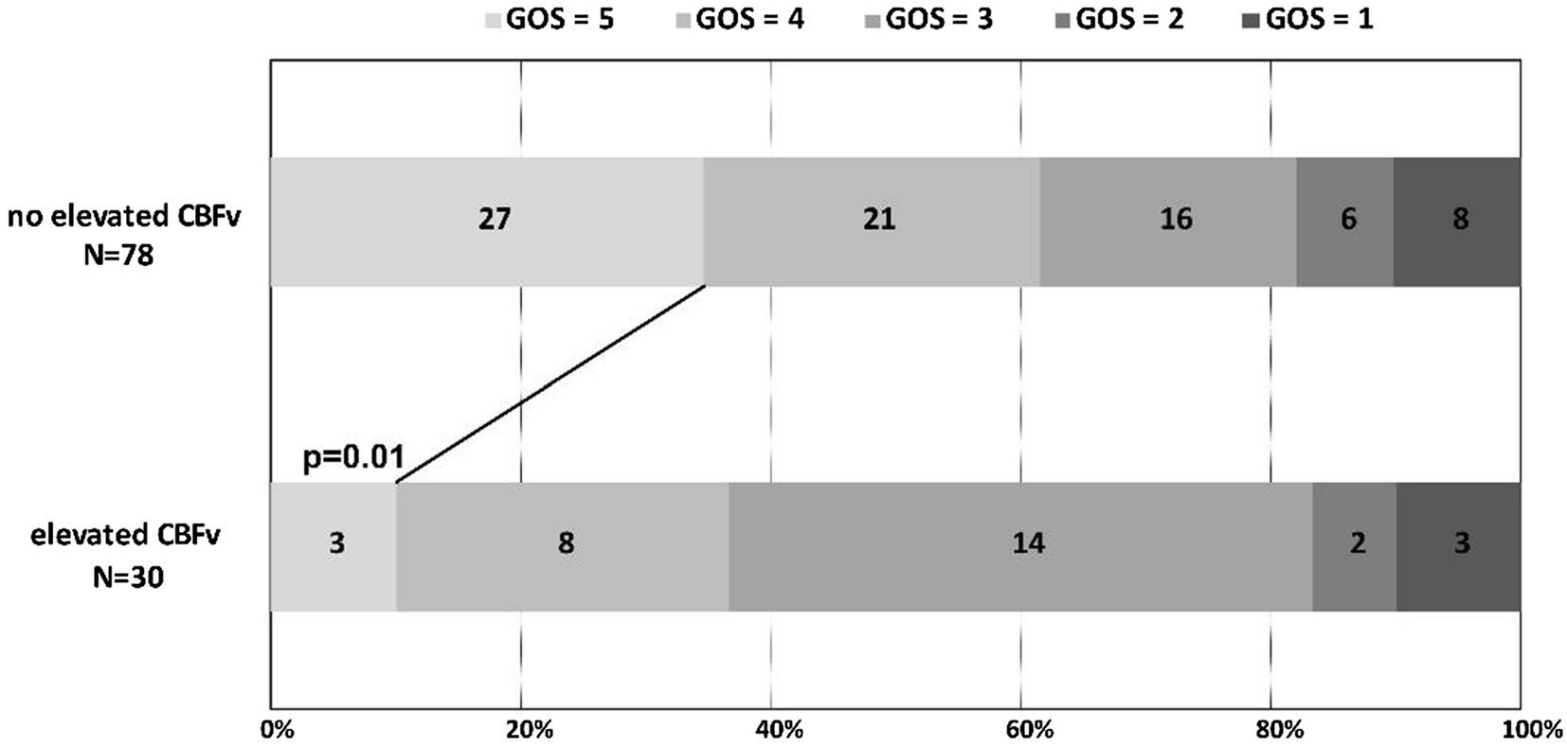
Glasgow Outcome Scale at discharge with elevated CBF versus no elevated CBF. Illustration of the proportion of patients with elevated cerebral blood flow velocity (n = 30) and no/normal elevated cerebral blood flow velocity (n = 78). Favorable functional outcome was defined as GOS = 5, unfavorable functional outcome as GOS = 1–4. *P* values were calculated for the comparison of unfavorable functional outcome among patients elevated and normal CBFv (*p* = 0.01). Abbreviations: *CBFv* cerebral blood flow velocity, *GOS* Glasgow outcome scale (range, 5 no or mild deficit, to 1, death).

ROC-analysis identified a NLR of 20.9 as the best cut-off threshold on admission to discriminate between increased CBFv within hospital stay (AUC = 0.713; *p* < 0.001, Youden’s index = 0.441; sensitivity, 63.3%; specificity, 80.8%; elevated CBFv: NLR ≥ 20.9 19/30[63.3%] vs. NLR < 20.9 15/78 [19.2%]; *p* < 0.001; Fig. *2*). These patients showed an unfavorable outcome (GOS 1–4) at discharge (elevated CBFv 27/30 [90.0%] vs. normal CBFv 51/78 [65.4%]; p = 0.01, Fig. 3).

## Discussion

There are three major findings from this cohort study on CABM-patients: (1) We identified elevated NLR on admission to be associated with increased CBFv within hospital stay. (2) ROC-analysis revealed a NLR of 20.9 as the best cut-off threshold on admission to discriminate between increased CBFv within hospital stay. (3) These patients showed an unfavorable outcome (GOS 1–4) at discharge.

According to published data^21,22^ NLR represents a promising parameter in the differential diagnosis of acute bacterial meningitis predominantly in patients aged 15 and older. As this dataset does not provide CSF counts on lymphocytes and neutrophils we are unable to reproduce these findings.

Cerebral vasospasms represent a devastating complication in acute bacterial meningitis contributing to an unfavorable functional outcome^10,31^. Our results imply that patients with an increased NLR on admission may yield a potential risk to develop an elevated CBFv which contributes to a potentially severe course of disease. Of 108 patients 30 (27.8%) developed increased cerebral blood flow velocity within hospital stay due to infection with *S. pneumoniae* in 70% in agreement of published data^31,32^. Among these patients ischemic complications were observed in 21.1%. TCD was performed routinely whereas cerebral imaging was performed according to clinical signs and symptoms. As not every patient with increased cerebral blood flow velocity underwent CT/MRI-imaging subclinical strokes may remained undetected. Further, increased flow velocities do not necessarily cause impending ischemic complications^26^.

In our cohort of patients with community acquired bacterial meningitis we identified a predictive NLR-cut off level to be ≥ 20.9. This cut-off value is increased compared to published data for e.g. prediction of ICH after endovascular thrombectomy in acute ischemic stroke^33^.

Unexpectedly and contrary to published data^34,35^ we did not identify dexamethasone administration to be independently associated with increased cerebral blood flow velocity in multivariate analysis. Prior to the wide spread use of adjunctive steroids in bacterial meningitis^36,37^ cerebral vascular alterations were devastating but common complications. Reasons may be cerebral vasculitis, septic emboli, intraarterial thrombosis or disseminated intravascular coagulation^35,38–40^. Thus specific anti-inflammatory regimes are desirable^41,42^ beyond adjuvant corticosteroid treatment which has been proven beneficial on case fatality rates in adult patients with pneumococcal meningitis but only in high-income countries^37^.

In acute intracerebral haemorrhage^14^, subarachnoid haemorrhage^43^ and ischemic stroke^44^ elevated NLR-levels were linked to unfavorable functional outcome. This could be shown by our data only in univariate testing (GOS 3 [3, 4] vs. 4 [3–5]; p = 0.028, mRS 4 [2–5] vs. 1 [1–4]; *p* = 0.020). Regarding long-term-outcome we did not identify significant differences. Reasons for this finding may be the small cohort size. According to recently published data, NLR represents a promising marker in stroke for the prediction of symptomatic hemorrhagic transformation (sHT) undergoing revascularization^45^ and cerebral edema after reperfusion therapy^46^.

Taken together, increased cerebral blood flow velocity is associated with neuroinflammation and represents a devastating complication in bacterial meningitis. NLR is an easy accessible and cost-effective biomarker that has proven its prognostic value in several conditions among cerebrovascular^14,47^ as well as infectious diseases^19,48^ and also in the differential diagnosis between viral and bacterial meningitis^21,22^. As a reliable parameter for the general immune response to various stimuli, NLR does not represent an inflammatory marker within a specific entity. Therefore, it remains unclear whether increased NLR-levels on admission reflect the state of bacterial infections or if high NLR values represent an independent surrogate to predict a severe course of the disease with intracranial vascular alterations^34^ as a devastating complication in bacterial meningitis. Future studies should investigate whether elevated NLR-levels in CABM-patients help to identify patients suitable for immune modulating treatments.

Our study has several limitations. First of all the small cohort size comprising only 108 patients weakens our data. It lacks a prospective and multi-center design. Except for admission laboratory data were not available every day within hospital stay. Bloodwork and CSF stains were done as required by clinical practice and not by scheduled timing. Further assessment of CBFv was conducted by different examiners within the inclusion period. As critical care procedures improved within the last years this may impose bias to the reported data. Finally, above findings do not provide any mechanistic evidence confirmed by specific blood sampling analyses or detailed immunoprofiling, and therefore depict a phenomenological finding.

## Conclusions

Among CABM-patients intracranial vascular complications are a risk factor for unfavorable outcome at discharge. Elevated NLR is independently associated with high CBFv and may be useful in predicting patients' prognosis. Future studies should investigate whether the impact of NLR represents an independent clinical implication or a preexisting comorbidity.

## Supplementary Information

Supplementary Table 1.

Supplementary Table 2.

## Data Availability

All data generated or analysed during this study are included in this published article.
