# Supplementary material for: Neutrophil-to-lymphocyte ratio is associated with increased cerebral blood flow velocity in acute bacterial meningitis
Source: Sci Rep. 2021 May 31;11:11383. doi: 10.1038/s41598-021-90816-0 (PMC8166920; doi:10.1038/s41598-021-90816-0)
Supplement: Supplementary file 1 — Supplementary Table 1. [file 41598_2021_90816_MOESM1_ESM.docx]

**Neutrophil-to-lymphocyte ratio is associated with increased**

**cerebral blood flow velocity in acute bacterial meningitis**

Antje Giede-Jeppe*^1^, MD, Selim Atay^1^, Julia Koehn^1^, MD, Anne Mrochen^1^, MD, Hannes Luecking^2^, MD, Philip Hoelter^2^  MD, Bastian Volbers^1^, MD, Hagen B. Huttner^3^, MD, PHD, Lena Hueske^4*#^, MD and Tobias Bobinger^1*#^, MD.

^1^ Department of Neurology, ^2^ Department of Neuroradiology; University of Erlangen-

Nuremberg, ^3^ Department of Neurology; University of Gießen, ^4^ Neurological Hospital for Parkinson's disease, Beelitz-Heilstaetten, Germany, ^#^ contributed equally.

**Supplementary table 1:** **Baseline Characteristics, Laboratory Data, In-hospital Measures and Outcome Parameters for all patients with community-acquired bacterial meningitis.**

| **Meningitis** | **(N=108)** |
| --- | --- |
| Age [yrs] # | 59 + 16 |
| Gender[♀]* | 52 (48.1%) |
| ***Prior medical history*** | |
| Premorbid mRS‡ | 0 (0-1) |
| Hypertension* | 58 (53.7%) |
| Malignoma* | 20 (18.5%) |
| Cardiac insufficiency* | 16 (14.8%) |
| Immunosuppression* | 14 (13.0%) |
| ***Admission status*** | |
| Neurologic deficit* | 30 (27.8%) |
| Glasgow Coma Scale (GCS)‡ | 14 (11-15) |
| Length of ventilation(LOV)[h]‡ | 9 (0-245) |
| Fever* | 62 (57.4%) |
| Meningism* | 69 (63.9%) |
| Headache* | 56 (51.9%) |
| Dexamethasone on admission* | 80 (74.1%) |
| Stay on Neurointensive Care Unit* | 103 (95.4%) |
| Osmotherapy* | 19 (17.6%) |
| Catecholamine therapy* | 54 (50.0%) |
| Intraventricular antibiotics* | 1 (0.9%) |
| Nimodipine therapy* | 26 (24.1%) |
| ***Laboratory values on admission*** | |
| *First spinal tap* |  |
| Leucocytes[x 10^6^/L]‡ | 1160 (187-5894) |
| Erythrocytes[x 10^6^/L] ‡ | 37 (2-275) |
| Proteine[g/L]‡ | 1.565 (0.681-3.387) |
| Glucose[mmol/L]‡ | 2.2 (0.1-3.4) |
| Lactate [mmol/L]‡ | 8.4 (3.9-15.5) |
| Causative pathogen identified by blood* | 48 (44.4%) |
| Causative pathogen identified by CSF* | 79 (73.1%) |
| Causative pathogen identified by CSF via PCR* | 42 (38.9%) |
| No causative pathogen identified* | 18 (16.7%) |
| *causative Pathogen:* |  |
| *S. pneumoniae** | 42 (38.9%) |
| *Group B Streptococcus** | 6 (5.6%) |
| *Group Non B Streptococcus** | 8 (7.4%) |
| *S. aureus** | 7 (6.5%) |
| *S. aureus coagulase(-)** | 4 (3.7%) |
| *N. meningitidis** | 6 (5.6%) |
| *L. monocytogenes** | 4 (3.7%) |
| *H. influenzae** | 4 (3.7%) |
| *E.coli** | 2 (1.9%) |
| *B. burgdorferi** | 2 (1.9%) |
| *B. hinzii** | 1 (0.9%) |
| *P. aeruginosa** | 1 (0.9%) |
| *M. tuberculosis** | 1 (0.9%) |
| *C. septicum** | 1 (0.9%) |
| *N. farcinica** | 1 (0.9%) |
| **Serum** |  |
| Neutrophil-Lymphocyte-Ratio‡ | 14.5 (6.7-23.1) |
| Hemoglobin[mmol/L]# | 7.5 + 1.0 |
| Leucocytes [10^9/L]# | 15.5 + 6.6 |
| Thrombocytes[10^9/L]# | 200 + 91 |
| Granulocytes[10^9/L]‡ | 13.5 (9.0-16.4) |
| Lymphocytes[10^9/L]‡ | 0.9 (0.6-1.2) |
| Monocytes[10^9/L]‡ | 0.8 (0.4-1.3) |
| C-reactive protein[mg/L]‡ | 219.8 (115.0-306.0) |
| Procalcitonin[µg/L]‡ | 2.9 (0.6-11.1) |
| Troponin[µg/L]‡ | 0.04 (0.01-0.12) |
| ***In-hospital measures*** | |
| Temperature [°C]‡ | 38.7 (38.0-39.3) |
| Sepsis* | 47 (43.5%) |
| Increased cerebral blood flow velocity* | 30 (27.8%) |
| Development of cbfv after admission[d]‡ | 4 (3-5) |
| ***Radiological Data*** | |
| *First CT on admission* | |
| Abscess* | 3 (2.8%) |
| Ischemia* | 4 (3.7%) |
| Obstructive hydrocephalus* | 5 (4.6%) |
| *CT within hospital stay- follow up (only 57/108 patients received more than one cerebral imaging)* | |
| Abscess* | 6/57 (10.5%) |
| Ischemia* | 12/57 (21.1%) |
| Obstructive hydrocephalus* | 4/57 (7.0%) |
| ***Discharge status*** | |
| Neurologic deficit at discharge* | 31 (28.7%) |
| Glasgow Outcome Scale (GOS)‡ | 4 (3-5) |
| Length of stay [d]‡ | 16 (10-23) |
| Length of ventilation‡ | 9 (0-245) |

‡= median (IQR), * = No. (%), # = mean + standard deviation.

Abbreviations: CBFv, cerebral blood flow velocity; GOS, Glasgow outcome scale (range, 5 no or mild deficit, to 1, death); mRS, modified Rankin Scale (range 0, no deficit, to 6, death); NLR, Neutrophil-to-Lymphocyte ratio; IQR, interquartile range; CSF, Cerebrospinal fluid.
