# Supplementary material for: Neutrophil-to-lymphocyte ratio is associated with increased cerebral blood flow velocity in acute bacterial meningitis
Source: Sci Rep. 2021 May 31;11:11383. doi: 10.1038/s41598-021-90816-0 (PMC8166920; doi:10.1038/s41598-021-90816-0)
Supplement: Supplementary file 2 — Supplementary Table 2. [file 41598_2021_90816_MOESM2_ESM.docx]

**Neutrophil-to-lymphocyte ratio is associated with increased**

**cerebral blood flow velocity in acute bacterial meningitis**

Antje Giede-Jeppe*^1^, MD, Selim Atay^1^, Julia Koehn^1^, MD, Anne Mrochen^1^, MD, Hannes Luecking^2^, MD, Philip Hoelter^2^  MD, Bastian Volbers^1^, MD, Hagen B. Huttner^3^, MD, PHD, Lena Hueske^4*#^, MD and Tobias Bobinger^1*#^, MD.

^1^ Department of Neurology, ^2^ Department of Neuroradiology; University of Erlangen-

Nuremberg, ^3^ Department of Neurology; University of Gießen, ^4^ Neurological Hospital for Parkinson's disease, Beelitz-Heilstaetten, Germany,

^#^ contributed equally.

**Supplementary table 2:** **Ischemic lesions**

| **Variable** | **Gender** | **Age** | **Pathogen** | **Vasospasm** | **Initial neuroimaging at admission** | **Repeat neuroimaging at time of delayed cerebral ischemia** | **Lesion volume** | **GOS discharge** |
| --- | --- | --- | --- | --- | --- | --- | --- | --- |
| **Patient 1** | Female | 49 | *S. hämolyticus* | + | CT: acute bilateral anterior and right middle cerebral artery infarction | CT: acute bilateral anterior and middle cerebral artery infarction | 9 ml (10ml) | 3 |
| **Patient 2** | Female | 38 | *M. tubercoulosis* | - | CT: multiple acute infarcts in bilateral hemispheres, e.g. septic embolism | - | Imaging data not available anymore | 1 |
| **Patient 3** | Male | *76* | *C. septicum* | *+* | CT: subacute, hemorrhagic transformed left middle cerebral artery infarction | *-* | *55 ml* | *1* |
| **Patient 4** | Female | 62 | *S. pneumoniae* | + | CT: acute frontal cortical/subcortikal infarcts | CT: acute bifrontal cortical/subcortikal infarcts | 11 ml (16ml) | 4 |
| **Patient 5** | Male | 64 | *S. pneumoniae* | - |  | CT: acute stroke in bilateral, in left thalamus and bilateral cerebellar, e.g. embolism | Imaging data not available anymore | 2 |
| **Patient 6** | Female | 58 | *S. pneumoniae* | - |  | Subacute stroke in right basal ganglia | Imaging data not available anymore | 2 |
| **Patient 7** | Male | 39 | *S. pneumoniae* | + |  | CT: multiple acute infarcts in bilateral hemispheres | 20ml | 3 |
| **Patient 8** | Male | 32 | *S. pneumoniae* | + |  | MRI: acute stroke in right hemisphere (e.g. due to cerebral vasospasm) | 5 ml | 4 |
| **Patient 9** | *Female* | *50* | *S. epidermidis* | *+* |  | CT: acute left anterior cerebral artery infarcts, e.g. due to cerebral vasospasm | *21 ml* | *3* |
| **Patient 10** | Male | 25 | *S. pneumoniae* | + |  | MRI: multiple acute bilateral anterior and middle cerebral artery infarction, e.g.septic embolism. | 2 ml | 4 |
| **Patient 11** | Female | 72 | *E. coli* | + |  | CT: subacute stroke in the left inferior gyrus frontalis | 1 ml | 3 |
| **Patient 12** | Male | 45 | *S. pneumonia* | + |  | CT: acute right anterior and middle cerebral artery infarcts (e.g. due to cerebral vasospasm) | 29 ml | 3 |
| **Patient 13** | Male | 57 | *F. nucleatum* | + |  | CT: Multiple acute infarcts in bilateral hemispheres and brain stem e.g. septic Embolism | 2 ml | 1 |
| **Patient 14** | Female | 66 | *N. farcinica* | - |  | CT: Multiple acute infarcts in bilateral hemispheres e.g. septic Embolism | 3 ml | 1 |

Abbreviations: CT, computed tomography; GOS, Glasgow outcome scale (range, 5 no or mild deficit, to 1, death), MRI, magnetic resonance imaging.
